# Supplementary material for: The Association between Dietary Patterns and Pre-Pregnancy BMI with Gestational Weight Gain: The “Born in Shenyang” Cohort
Source: Nutrients. 2022 Jun 20;14(12):2551. doi: 10.3390/nu14122551 (PMC9229543; doi:10.3390/nu14122551)
Supplement: Supplementary file 1 [file nutrients-14-02551-s001.zip › nutrients-1749147-supplementary.pdf]

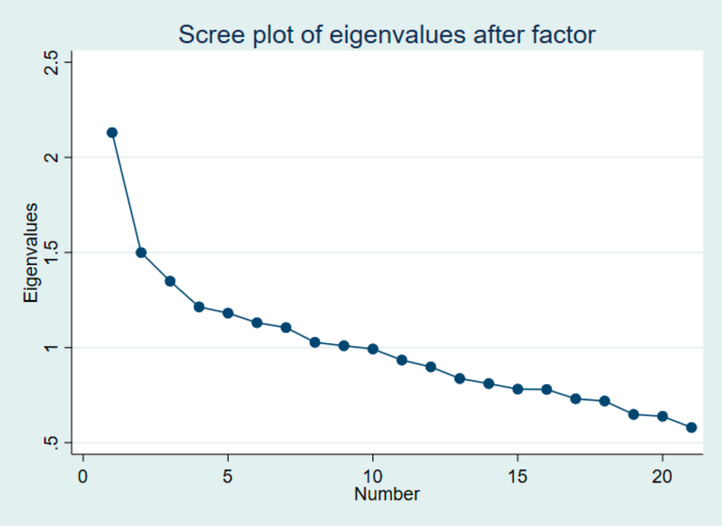

Figure S1. Scree plot from exploratory factor analysis.

Table S1. Complete list of the 21 food groups.

Food group	Food items
Rice	Rice, rice flour
Wheat flour and products	Noodle, bread, bun, flour, steamed twisted roll
Whole grains	Brown rice, millet, sorghum, black rice
Tubers	Potatoes, sweet potatoes,
Red meat	Pork, pork ribs, pork fat, beef, lamb, donkey, rabbit
Poultry	Chicken, duck, goose
Organ meats	Liver, kidney, blood, large intestine
Freshwater fish	Carp, squid, grass carp, chub, catfish
Marine fish	Hair tail, yellow croaker, squid, herring, striped bass
Shrimps, crabs and mussels	Shrimp, crab, scallop, oyster
Seaweed	Fresh or dried seaweed
Milk	Cow milk, goat milk
Dairy products	Yogurt, cheese
Eggs	Egg, duck egg, goose egg
Beans and bean products	Broad bean, soy, tofu, dried tofu, soy milk
Fruits	Apple, pear, orange, banana, grape, peach, watermelon, cherry, jujube
Vegetables	Spinach, cabbage, Chinese cabbage, celery, rape, oil wheat, western lettuce, chives, tomato, cucumber, carrot, winter melon, onion, green paper, broccoli, mushroom
Nuts	Peanut, walnut, sesame, cashew, almond, sunflower seed, pistachio
Pastry and candy	Jelly, jam, chocolate, honey, sugar, candy, mooncake
Fried foods	Chips, French fries, fritters, fried chickens, fried fish
Sweet beverages	Coke, soda, fruit juice, soft drinks

Table S2. Characteristics of participants included and excluded in the present analysis

Characteristics	Participants, n (%)		
	**Included**	**Excluded**	***P* value**
Age at enrollments (years)			<0.01
<25	60 (5.9)	12 (4.4)	
25-29	450 (43.9)	96 (35.6)	
30-34	358 (34.9)	92 (34.1)	
≥35	158 (15.4)	70 (25.9)	
Ethnicity			<0.01
Han	855 (83.3)	193 (71.5)	
Others	171 (16.7)	77 (28.5)	
Educational attainment			0.13
High school or below	241 (23.5)	66 (28.2)	
College or above	785 (76.5)	168 (71.8)	
Household income per year, CNY			0.37
<50,000	544 (53.0)	482 (47.0)	
≥50,000	126 (48.5)	255 (45.7)	
Parity			0.38
Primipara	795 (77.5)	175 (75.8)	
Multipara	231 (22.5)	59 (25.2)	
Pre-pregnancy BMI category, kg/m^2			0.21
<18.5	134 (13.1)	32 (13.7)	
18.5-<25.0	700 (68.2)	147 (62.8)	
≥25.0	192 (18.7)	55 (23.5)	
Smoking during or before pregnancy			<0.01
No	1020 (99.4)	232 (85.9)	
Yes	6 (0.6)	38 (14.7)	
Physical activity status during pregnancy, MET-hour/week			0.01
<100	266 (25.9)	96 (35.6)	
100-200	556 (54.2)	124 (45.9)	
>200	204 (19.9)	50 (18.5)	
Energy intake, kcal/d			0.78
<2100	619 (60.3)	407 (40.0)	
≥2100	74 (61.7)	46 (38.3)	

CNY: Chinese Yuan, BMI: body mass index, MET: metabolic equivalent

Table S3. Crude and adjusted odds ratios for being EGWG by the quartiles of dietary pattern scores, further adjusted for pre-pregnancy BMI status.

Dietary patterns	Risk of Being EGWG (ref. = Non-EGWG, n = 1026)				
	**Q1 Reference**	**Q2 OR (95%CI)**	**Q3 OR (95%CI)**	**Q4 OR (95%CI)**	***P* for trend**
Traditional pattern	1.00	1.06 (0.73,1.54)	1.62 (1.10,2.40)	1.67 (1.04,2.67)	0.02
Sweet foods pattern	1.00	0.66 (0.46,0.95)	1.03 (0.71,1.49)	0.94 (0.65,1.37)	0.79
High protein pattern	1.00	0.66 (0.46,0.96)	0.56 (0.38,0.81)	0.67 (0.46,0.99)	0.10
Milk-nut-seafood pattern	1.00	0.83 (0.58,1.20)	0.94 (0.65,1.37)	1.04 (0.70,1.53)	0.64

Adjusted for other dietary pattern score, age, parity, family income, education level, ethnicity, smoking status, total energy intake per day, physical activity status per week, pre-pregnancy BMI status.

EGWG: excess gestational weight gain, BMI: body mass index, Q1: quartile 1; Q2: quartile 2; Q3: quartile 3; Q4: quartile 4; OR: odd ratio; CI: confidence interval.

Table S4. Associations of maternal dietary pattern scores, in quartiles, with gestational weight gain rate

Dietary patterns	Gestational Weight Gain Rate (kg/w, n = 1026)				
	**Q1 Reference**	**Q2 β (95%CI)**	**Q3 β (95%CI)**	**Q4 β (95%CI)**	***P* for trend**
Traditional pattern	1.00	0.01 (-0.01, 0.04)	0.02 (-0.01, 0.04)	0.02 (-0.01, 0.05)	0.28
Sweet foods pattern	1.00	-0.02 (-0.04, 0.01)	0.01 (-0.01, 0.03)	-0.01 (-0.03, 0.02)	0.85
High protein pattern	1.00	-0.03 (-0.05, -0.01)	-0.03 (-0.06, -0.01)	-0.03 (-0.06, -0.01)	0.04
Milk-nut-seafood pattern	1.00	-0.02 (-0.04, 0.00)	-0.03 (-0.05, 0.00)	-0.01 (-0.04, 0.01)	0.45

Adjusted for other dietary pattern score, age, parity, family income, education level, ethnicity, smoking status, total energy intake per day, physical activity status per week, pre-pregnancy BMI status.

BMI: body mass index, Q1: quartile 1; Q2: quartile 2; Q3: quartile 3; Q4: quartile 4; CI: confidence interval.
